# Case report: Major pathologic response induced by neoadjuvant treatment using BRAF and MEK inhibitors in a patient with stage IIIA lung adenocarcinoma harboring BRAF V600E-mutation

**DOI:** 10.3389/fonc.2022.961539

**Published:** 2022-08-08

**Authors:** Chaoyuan Liu, Min Lu, Yang Yang, Xiang Wang, Fang Ma, Xianling Liu

**Affiliations:** ^1^ Department of Oncology, The Second Xiangya Hospital, Central South University, Changsha, China; ^2^ Department of Pathology, The Second Xiangya Hospital, Central South University, Changsha, China; ^3^ Department of Thoracic surgery, The Second Xiangya Hospital, Central South University, Changsha, China

**Keywords:** lung cancer, BRAF V600E mutation, neoadjuvant, targeted therapy, major pathologic response

## Abstract

Targeted therapy has achieved great success in advanced non-small lung cancer (NSCLC) with driver genes, and neoadjuvant-targeted therapy is increasingly being investigated. Although neoadjuvant-targeted therapy with EGFR-TKI and ALK-TKI showed good efficacy, there is no report of neoadjuvant-targeted therapy to BRAF V600E mutation on NSCLC so far. Here, we report the first case of a successful neoadjuvant-targeted therapy with BRAF and MEK inhibitors followed by radical surgical excision with major pathologic response (MPR) in a patient with stage IIIA lung adenocarcinoma (LUAD) harboring BRAF V600E mutation. The case informs us that targeted therapy with BRAF and MEK inhibitors could be administrated as a neoadjuvant strategy for selected cases of NSCLC harboring BRAF V600E mutation.

## Introduction

The emergence of targeted therapy and immunotherapy has brought about revolutionary changes in the treatment of non-small lung cancer (NSCLC). As for NSCLC with driver genes, the efficacy of immunotherapy is poor; targeted therapy is still the mainstream treatment option. The clinical success of targeted therapy in patients with advanced NSCLC has also prompted an assessment of their efficacy in the neoadjuvant setting. BRAF mutation is recognized as driver mutation in NSCLC and has been reported in 1%–5% of NSCLC cases, with the BRAF V600E mutation present in 50% of these cases (1). Due to the rarity of this mutation, the neoadjuvant treatment to BRAF V600E mutation is less investigated compared with EGFR-TKI and ALK-TKI. Published data on the efficacy of neoadjuvant-targeted therapy to BRAF V600E mutation in the treatment of malignancy is limited, especially NSCLC. Herein, we report the first rare case of successful neoadjuvant-targeted therapy with BRAF and MEK inhibitors followed by radical surgical excision with major pathologic response in a patient with stage IIIA lung adenocarcinoma harboring a BRAF V600E-mutant.

## Case report

**Figure 1:**
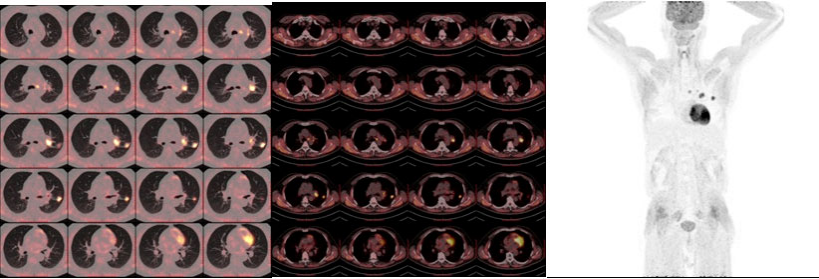
PET-CT at baseline 1 month before dabrafenib/trametinib treatment. PET-CT scan of the chest at baseline prior to dabrafenib plus trametinib therapy demonstrated a nodule in the upper left lung with elevated uptake of 18F-fluorodeoxyglucose (FDG), accompanied by enlargement of multiple lymph nodes of the left lung hilum as well as mediastinum (4L, 5, 6). No other lesions with elevated uptake of 18F-FDG were found.

A non-smoking 56-year-old man was presented with cough in September 2021; family history, physical examination, and laboratory studies have no positive findings. PET-CT revealed a nodule in the upper left lung (23 mm × 19 mm), accompanied by enlargement of multiple lymph nodes of the left lung hilum as well as mediastinum (4L, 5, 6) (**Figure 1**). Magnetic resonance imaging (MRI) of the brain was negative for metastatic disease. Computed tomography (CT)-guided percutaneous transthoracic needle biopsy of the nodule was performed, and pathological analysis and multiple-gene test (26 genes panel) showed lung adenocarcinoma with BRAF V600E (abundance 24.39%) and a TP53 mutation (abundance 21.16%); the sample was negative for oncogenic alterations in EGFR, ALK, ROS1, KRAS, MET, and RET. The PD-L1 tumor proportion score (TPS) was 90% (Dako 22C3). Therefore, the patient was diagnosed with an AJCC 8th stage IIIA (cT1cN2M0) primary left lung adenocarcinoma harboring BRAF V600E and PD-L1 TPS 90%+ after a complete initial evaluation.

**Figure 2:**
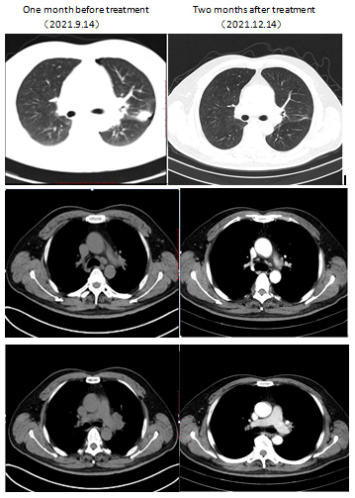
Computed tomography imaging showed initial response to combination of dabrafenib and trametinib. Left column: CT scan of the chest at baseline 1 month prior to dabrafenib plus trametinib therapy demonstrated a nodule in the upper left lung (lung window) accompanied by enlargement of multiple lymph nodes of the left lung hilum as well as mediastinum (mediastinal window). Right column: CT scan at 2 months after therapy demonstrated a marked response with a significant decrease in the nodule, left lung hilum, and mediastinum.

**Figure 3:**
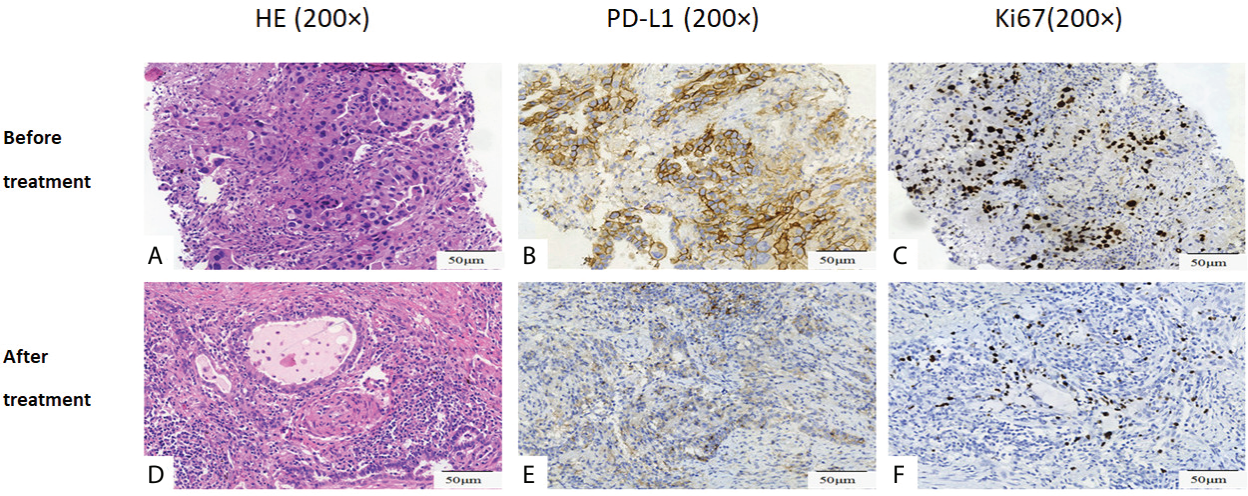
Histopathological assessment of tumor regression. **(A-C)** Pathological picture prior to dabrafenib plus trametinib therapy. **(D-F)** Pathological picture post dabrafenib plus trametinib therapy. **(A)** demonstrated moderately differentiated adenocarcinomas. **(D)** demonstrated marked necrosis and massive benign fibrous tissue proliferation with very few residual tumor cells in resected tumor. **(B)** and **(E)** showed PD-L1 90% **(B)** and 5% **(E)** before and after dabrafenib plus trametinib therapy. **(C)** and **(F)** showed Ki67 35%**(C)** and 1%**(F)** before and after dabrafenib plus trametinib therapy. Original magnification: **(A–F)**: ×200.

**Figure 4:**
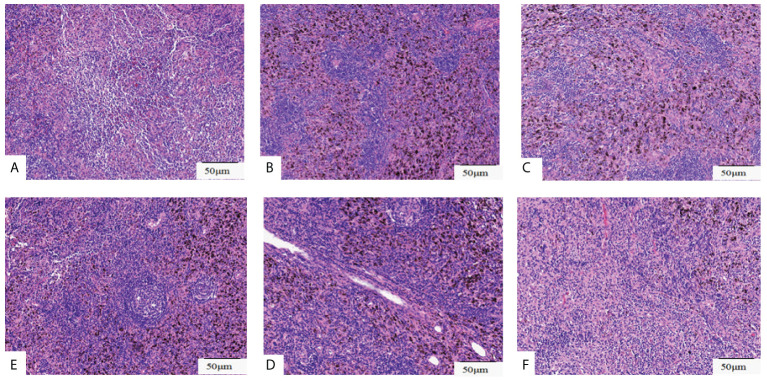
Histopathological assessment of lymph nodes resected. Pathological pictures of lymph nodes station 4L**(A)**, station 5**(B)**, station 7**(C)**, station10 **(D)**, station 11**(E)**, station 12**(F)**. Original magnification: **(A-F)**: ×200.

**Figure 5:**
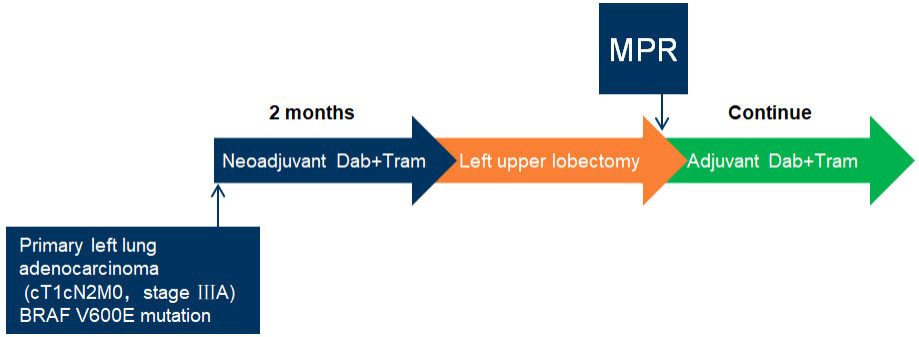
Brief summary of the therapeutic process.

After diagnosis, the patient initiated treatment with orally dabrafenib 150 mg twice daily in combination with orally trametinib 2 mg daily. Five days later, the patient experienced recurrent episodes of pyrexia which were due to community-acquired pneumonia and maybe therapy-related. The pyrexia was managed by the use of antibiotics and temporary drug dose interruption; the pyrexia resolved 10 days after the use of antibiotics and drug dose interruption. Chest CT demonstrated significant improvement of pneumonia; the patient restarted the bi-targeted therapy with the same dose 2 weeks after the interruption. The cough improved quickly and finally resolved. Two months later, at the first radiological assessment, his chest CT scan showed partial remission (PR) (**Figure 2**). The patient underwent robotic surgery of left upper lobectomy and systematic lymphatic dissection was performed, the surgery procedure was smooth, and the patient was discharged without complications 4 days after the operation. Postoperative pathological analysis showed major pathologic response for the tumor (**Figure 3**), and all 14 lymph nodes resected (station 4L, 5, 7, 10, 11, 12) (**Figure 4**) were negative. Postoperative adjuvant treatment with dabrafenib and trametinib was prescribed 1 month after surgery. The therapeutic process is shown in **Figure 5**. During the disease course, he only experienced aforementioned pyrexia, which lasted 10 days and resolved with use of antibiotics and the temporary interruption of bi-targeted therapy. No other toxic side effects were observed. The patient currently remains in good condition and complete remission at 3 months after surgery. The case presented here supports the use of neoadjuvant treatment with BRAF inhibitors in local advanced non-small lung cancer with BRAF V600E mutation. Our study received approval from the ethics committee of the second Xiangya Hospital, Central South University. The patient also provided written informed consent to publish this case report details.

## Discussion

BRAF mutations are detected in 3%–8% of LUAD (2). The BRAF V600E mutation test is recommended for all patients with newly diagnosed advanced LUAD (3). Based on the results of a phase II clinical trial for patients with BRAF V600E–mutated NSCLC (NCT01336634), the U.S. Food and Drug Administration (FDA) has approved the combination therapy of BRAF inhibitor dabrafenib and the MEK1/2 inhibitor trametinib for treatment of advanced NSCLC patients with BRAF-V600E mutation, regardless of previous therapies (1, 4–6). In the trial, in cohorts for pretreated and treatment-naive patients, the ORR of the combination therapy was 63% and 64% and the median PFS was 9.7 and 10.9 months; the recent updated 5-year overall survival rates were 19% and 22%, respectively.

Neoadjuvant-targeted therapy with EGFR-TKI and ALK-TKI has got promising preliminary results (7, 8). Like neoadjuvant EGFR-TKI and ALK-TKI therapy, the combination therapy of dabrafenib and trametinib also showed promising potential for neoadjuvant therapy in a series of malignancy, such as melanoma, anaplastic thyroid carcinoma, and papillary craniopharyngioma (9–12). However, no such case of successful neoadjuvant therapy with combination therapy of dabrafenib and trametinib has been reported in NSCLC so far.

Here we presented a case of stage IIIA (cT1cN2M0) primary left lung adenocarcinoma harboring BRAF V600E mutation. The patient had an excellent response to preoperative treatment with BRAF/MEK inhibitors and obtained MPR after surgery. To our knowledge, the MPR achieved with the use of BRAF/MEK inhibitors has not been previously reported in the literature of NSCLC. The impressive response suggests that presurgical targeted therapy may be combined with surgery to achieve long-term survival in NSCLC with BRAF V600E mutation. Moreover, immunohistochemical results demonstrated that the expression of both PD-L1 and Ki67 decreased significantly after the use of BRAF/MEK inhibitors. Ki67 is a well-known cell proliferation mark for many tumor types including NSCLC; its decrease suggested that BRAF/MEK inhibitors harmed the proliferation capacity of NSCLC cells. A study showed that PD-L1 in some tumor cells would be downregulated at the beginning of BRAF/MEK inhibition and be upregulated after acquiring resistance to the treatment (13). Another study demonstrated in human colon cancer cell endogenous or exogenous BRAF V600E mutant vs. wild-type BRAF could increase PD-L1 messenger RNA (mRNA) and protein expression that was attenuated by MEK inhibition (14). The change of PD-L1 expression in the present case is consistent with these studies.

The role of immunotherapy in BRAF-mutated NSCLC patients is not established; clinical evidence of immunotherapy efficacy in such patients is only retrospective. In a study, BRAF-mutant NSCLC is associated with a high level of PD-L1 expression (15); the present patient who has a high expression of PD-L1 with TPS 90%+ is consistent with the study. Several retrospective studies demonstrated that the efficacy of immunotherapy in BRAF-mutated NSCLC patients is comparable to those observed in the unselected NSCLC population (15–17). A multinational retrospective study demonstrated that the objective response rate (ORR) of NSCLC harboring a BRAF mutation treated with immune checkpoint inhibitor (ICI) monotherapy is only 24%; median PFS was only 3.1 months (18). ICI monotherapy for NSCLC with driver genes including BRAF V600E mutation is unsatisfactory, so it is widely recommended that NSCLC with driver gene mutation should receive targeted therapies and chemotherapy before considering immunotherapy as a single agent (4, 18). The Checkmate 816 trial indicated a significantly higher pathological complete response rate of neoadjuvant immunochemotherapy than of neoadjuvant chemotherapy alone (odds ratio, 13.9). The trial included NSCLC with stage IB to IIIA and excluded EGFR or ALK mutation; it did not exclude BRAF V600E mutation, but there is no report about the efficacy of neoadjuvant immunochemotherapy to the BRAF V600E mutation subgroup in the trial. Although there was a NSCLC patient harboring BRAF V600E mutation who achieved pathological complete remission (PCR) after neoadjuvant combination of the PD-1 antibody and chemotherapy in our department, there is no comparative study of the combination with ICI and chemotherapy vs. combination of bi-targeted therapy in advanced/metastatic NSCLC harboring BRAF V600E mutation so far. Thus, which combination is the most effective strategy as neoadjuvant therapy needs further investigation. Regardless, the success of the neoadjuvant bi-targeted therapy for the present patient demonstrated that the combination of dabrafenib and trametinib may be used as neoadjuvant therapy for potential resectable NSCLC with BRAF V600E mutation.

## Conclusion

Combination of BRAF and MEK inhibitors may be used as neoadjuvant therapy followed by surgical resection to improve the outcome of local advanced NSCLC patients harboring BRAF V600E mutation.

## Data availability statement

The raw data supporting the conclusions of this article will be made available by the authors, without undue reservation.

## Ethics statement

This study was reviewed and approved by the ethics committee and institutional review board of the Second Xiangya Hospital, Central South University. The patient provided his written informed consent to publish his case report.

## Author contributions

Main contribution: CL collected, analyzed, and interpreted the data and drafted the manuscript; ML and YY collected the materials and prepared the figures. XW performed the surgery and revised the final manuscript. FM and XL conceived and critically revised the final manuscript. All authors contributed to the article and approved the submitted version.

## Conflict of interest

The authors declare that the research was conducted in the absence of any commercial or financial relationships that could be construed as a potential conflict of interest.

## Publisher’s note

All claims expressed in this article are solely those of the authors and do not necessarily represent those of their affiliated organizations, or those of the publisher, the editors and the reviewers. Any product that may be evaluated in this article, or claim that may be made by its manufacturer, is not guaranteed or endorsed by the publisher.
